# A Paging Training Program for a Fourth-Year Internship Readiness Course

**DOI:** 10.15766/mep_2374-8265.11021

**Published:** 2020-11-13

**Authors:** Emily Cetrone, Kathryn Mutter, Kathryn Pedersen, Neeral Shah, James Martindale

**Affiliations:** 1 Resident, Department of Medicine, Brigham and Women's Hospital; 2 Assistant Professor, Department of Emergency Medicine, University of Virginia; 3 Surgical Trauma Burn Intensive Care Unit, University of Virginia; 4 Associate Professor of Medicine, Gastroenterology and Hepatology, University of Virginia; 5 Associate Professor of Medical Education Research, Director of Assessment, University of Virginia

**Keywords:** Mock Paging, Internship Preparation, Simulation, Communication Skills, Triage, Nurse/Nurse Practitioner, Physician, Communication Skills, Interprofessional Education, Leadership Development/Skills, Emergency Medicine, Internal Medicine, Case-Based Learning, Clinical Skills Assessment/OSCES

## Abstract

**Introduction:**

New medical interns face a steep learning curve as they must manage complex medical scenarios, many of which they have only seen before in a classroom setting. To ameliorate these challenges, medical schools are increasingly including courses designed to address the transition from student to doctor. One of the biggest challenges for new interns is learning to triage and manage nursing pages, so we designed a mock paging program incorporated within our fourth-year transitions course.

**Methods:**

We developed a database of clinical scenarios to occur via telephone between a nurse and a medicine intern. Throughout the 2-week course, these cases were administered to 40 fourth-year medical students by Master's level nursing students and nurse evaluators. The nurses used checklists to evaluate medical student management and communication, and at the end of the phone encounter students received immediate feedback. We used an observational prospective design, using a within subjects method with repeated measures.

**Results:**

Data from a total of 216 phone calls were analyzed for 36 students. No statistically significant improvement of checklist scores was observed. Substantial interrater reliability was observed for the four observed cases with a Fleiss-Kappa of .76. Student comments indicated the activity was helpful for preparing them to answer pages.

**Discussion:**

Our paging program offered students the chance to simulate being on call, as well as the opportunity to receive immediate feedback. It did not show improvement in checklists across time. Limitations included a small sample size and few common variables across the cases.

## Educational Objectives

By the end of this activity, learners will be able to:

1. Apply an effective approach to answering pages from registered nurses concerning patients under their care.
2. Demonstrate clear and effective communication on phone calls with registered nurses, including asking specific questions and using closed-loop communication.
3. Initiate the assessment and management of medical issues over the telephone.
4. Recognize acute scenarios that require prompt bedside assessment.

## Introduction

From the very first day, new interns face a steep learning curve. They must confront and manage complex scenarios, many of which they have only seen before in a classroom setting. Increasingly, more medical schools across the country are recognizing this deficit and instituting courses in the final year specifically designed to prepare learners for this transition from student to doctor.^1–3^ One of the key transitional skills identified by these courses is communication, since miscommunication has been noted repeatedly as a contributor to patient care errors and adverse events.^4^ Many factors are involved in these communication errors, including hierarchical differences, conflicting roles, role ambiguity,^5^ and a lack of training in this area in medical education.^6^ Furthermore, across the medical spectrum, a significant portion of communication in health care occurs via the telephone, including the exchange of important patient information and clinical decision-making. Despite this, there is still a paucity of training in this area.^7^

One particularly challenging aspect of becoming an intern is learning to manage pages, as this combines acute medical management with communication (frequently via telephone), time management, and triage skills. Among the aforementioned courses that prepare fourth-year medical students for intern year, several included curricula specifically designed to target the skill of managing and responding to pages.^1–3,8^ Most of these paging programs exist for students going into obstetrics and gynecology or surgery, with fewer for those going into internal medicine. There are also some differences in how the curricula are delivered, but for the most part the programs run in distinct blocks of time. For example, in one course, the paging content is delivered in discrete 2-hour sessions, during which students take turns receiving single pages from nurse practitioners.^2,9,10^

Our work expanded upon these existing paging programs. As mentioned, most of them, including those in *MedEdPORTAL*, occur over the course of a single session or day. While there are some obvious logistical benefits to this approach, we wanted the experience to be spread out over our existing 2-week course, in order to better simulate the cognitive load of intern year. There is something very specific to the stress and skill needed when pages come randomly during a block of time you are on call. In particular, we wanted to simulate one of the hardest parts of being a new intern, which is the challenge of having to appropriately triage pages. Though we were unable to perfectly encapsulate this with multiple pages coming in at once, receiving multiple of these pages over the 2 weeks allowed for spaced learning.

In order to address the course goals and create a unique educational curriculum, we developed our mock paging program between Master's level nursing students and nurses and fourth-year medical students during a fourth-year internship readiness course. We wanted to determine if this program increased medical student performance over the 2 weeks with post-paging feedback from the nurses/nursing students and if the program improved the medical students' self-reported preparation to answer pages.

## Methods

### Curricular Context

At the University of Virginia (UVA) School of Medicine, there was no existing formal training in the curricula for future medical interns on telephone-based communication, so we created this mock page activity to address deficit. We developed a database of case-based clinical scenarios with accompanying checklists that would occur via telephone between a nurse and a first-year medicine intern (Appendix A).

These cases were incorporated into the 2-week Preparation for Internship course, which was offered to fourth-year medical students in the Spring to provide the necessary skills to prepare students for intern year (e.g., electrolyte replacement, note writing, discharges, and management of acutely deteriorating patients) using simulation. Our paging program was woven into this course, which occurred after Match Day, after which students were divided into surgery, pediatric, obstetrics and gynecology, and acute care/medicine groups based upon their residency match. This paging program was only offered to the acute care/medicine group, but students in the pediatric, surgical, and obstetrics and gynecology groups took part in a different paging program.

The acute care/medicine group consisted of students who had matched into internal medicine, family medicine, and emergency medicine, as well as any who would be doing a medicine preliminary year, such as radiology, dermatology, and neurology. The research was deemed exempt from further review by the UVA Institutional Review Board. Students gave consent to include their data in the research.

### Materials Development

The cases and checklists (Appendix A) were developed by a team of a fourth-year medical student, an emergency medicine faculty member, an internal medicine faculty member, and a practicing nurse. These cases were developed after review of the intern handbook provided to UVA medical interns, and they were reviewed by the participating faculty. A pilot session occurred with a fourth-year medical student who was not participating in the acute care/medicine course. Based upon the feedback and observations of the pilot session, the cases and checklists were further refined for added clinical details and clarity of language.

### Facilitator Training

A key component of our program was the interaction between the medical student and nurse/nursing student. Prior to implementing our curriculum, the leaders of the course met with each of the nurses for a training session. During this session, the trainer reviewed the cases and checklists with the nurses, as well as the objectives of the course. The nursing students were Master's level, so both they and the nurses already had clinical experience and were instructed to handle the phone call as they would in a true hospital setting. For the debriefing, they were asked to mostly focus on specific examples where the medical student effectively or ineffectively communicated. Additionally, they were informed that there was no penalty for students asking questions concerning the clinical scenario beyond what was included in the checklist. For example, for case 1, the checklist focused on questions related to the diagnosis of pulmonary embolism, but it would be entirely appropriate for students to ask questions pertaining to possible acute coronary syndrome or gastrointestinal bleed. In subsequent years since the initial program, nurses who have participated in the program before have taken on the role of training other nurses/nursing students.

### Implementation

At the beginning of the course, there was a brief orientation for the paging program. During this session students were given a list of patients that they were covering, as well as some information about them that would be typical of a sign-out (Appendix B). The sign-outs included in Appendix B are organized and identified by case for reference purposes, but the patient scenarios were mixed up and organized by room number when given to medical students. The orientation also included an explanation of the paging program learning objectives, including the overall goal of creating a simulated environment. Students were instructed that when receiving a page, they should respond as if they were on a busy cross-cover night and therefore would have to take into consideration the level of urgency and triage the scenario appropriately. It was emphasized during the orientation session that students should also make clear on the phone about how quickly they would assess the patient at bedside. For example, if they were told about a patient that was hypotensive, they should immediately assess the patient if possible. In this simulated environment, the students should act as if they were managing another decompensating patient and therefore should start the initial assessment over the telephone, making clear that they will come to the bedside immediately following. They were also given their call schedule, which entailed several blocks of time over the 2 weeks.

Throughout the 2 weeks, students received a total of six pages at random times (from 7:00 am to 11:00 pm). The clinical scenarios concerned patients that the student was cross-covering for the shift. While on call, the medical student would receive a page (text message) from a nursing student or nurse (heretofore referred to as the evaluator) concerning one of the patients on this list. The page would identify the patient by room number and would give a brief one-liner. For example, one page read, “Urgent need action. 3134 w SOB, O2 sat 83%.” The page would end with the name of the evaluator sending the page and would provide a call-back number.

Once the medical student called, the evaluator would provide a brief clinical scenario that a medicine intern would commonly encounter. From there, the conversation proceeded in a way that was based on the student's responses and questions. The evaluator used a checklist (Appendix A) to evaluate the student during this call based on completeness, accuracy, and overall communication skills.

### Assessment

We used an observational prospective, repeated-measures design. A Friedman two-way non-parametric analysis of variance was used to determine if there were statistically significant differences in checklist performance by medical students across cases. Interrater reliability of checklists was determined by a Fleiss' Kappa statistic, with 10 Master's level nursing students listening to four prerecorded cases.

## Results

In the spring of 2017, there were 40 fourth-year medical students enrolled in the acute care/medicine cohort of the Preparation for Internship course. Ten Master's level nursing students and nurses participated as evaluators, using six unique cases with accompanying checklists. Data from a total of 216 phone calls were analyzed for 36 students. Data for four students were lost either due to an incomplete set of data or nonelection to participate. There was no statistically significant improvement of checklist scores observed over the curriculum. Substantial interrater reliability was observed for the four observed cases with a Fleiss-Kappa of .76. In addition to giving immediate feedback at the end of each phone call, the evaluators also recorded some comments on each checklist. Examples of comments included:

• “Said she would come see him and gave orders of meds/recheck blood pressure while she was on the way. Very comfortable on the phone. Consistently had closed-loop communication.”
• “Could work more on being organized and confidence.”
• “Great job! You hit all the highlights. Organized, composed. Called back in a timely manner being an urgent page.”

Students were asked for overall comments about the learning experience, and they expressed that overall, the activity was helpful. Select comments included:

• “I think the mock paging was very helpful.”
• “I found the paging exercise tremendously useful.”
• “It is helpful to practice thinking on the spot.”
• “Overall I found this exercise to be useful. It helped me be systematic in how I answered pages and collected relevant clinical information. It also helped me think through initial management over the phone before I could go and see the patient.”

Students were also asked for suggestions for improvement. Recommendations included more practice with briefer interactions, as well as practice with incorporating the electronic medical system into the scenario.

## Discussion

Our mock paging course helped fourth-year medical students feel more prepared in responding to pages from nurses. Through the mock page scenarios, students had the opportunity to practice closed-loop communication and acute medical management skills they would shortly need in intern year. In addition, they could get immediate feedback from their evaluator and participants had the chance to be on call, more closely approximating the intern experience and making this course different from most existing mock page programs.^1–3^

Our course did not require significant material resources, but it did require the personnel to implement it. We wanted our program to be interdisciplinary, so we chose to have Master's level nursing students and nurses serve as the evaluators. We felt this added value to the experiential learning, as the nursing students and nurses had a unique perspective to offer and the feedback would be more authentic. If this option were not available at an institution, others, including faculty or residents, could substitute for this role.

The challenge of having to appropriately triage pages is one of the hardest parts of being a new intern, which were unable to fully simulate through this activity. Because the six pages were spread out over two weeks and did not all come in at once, the students were instructed to manage the page as if they were in the role of an intern covering a busy service. For example, for the case concerning back pain the goal was to assess and manage the situation over the telephone, which would be more appropriate than seeing the patient on a busy cross-cover shift. Therefore, with our structure, the learner not only had to communicate effectively with another health care provider via telephone, they must triage and respond to an unanticipated call and decide how much of the assessment and management should be conducted via telephone.

With our study, we were not able to show that students improved on the checklist as they progressed through the six cases. There were a number of possible contributing factors. Firstly, our sample size was small, and 2 weeks was a limited amount of time in order to show progress across a small number of cases. More significantly, our checklists had very few common variables across cases, as they assessed both medical knowledge and communication skills. Therefore, each checklist was unique. This limited our ability to track improvement in any one area across time. Instructors should be aware that these checklists were not ideal tools to assess for improvement. We still found value in the checklists, however, because they served as excellent tools for the evaluators to provide immediate feedback to the students. Over time, they may also be valuable in determining additional needed training and could easily be adapted to assess different skills.

One additional consideration is that although closed-loop communication and goal-directed questioning were the only communication strategies used in this initial rollout, in further iterations since 2017 a more standardized format of introduction, situation, background assessment, recommendation (ISBAR)^11^ has been taught to the medical students. ISBAR was taught at the beginning of the preparation course so that students may then use this strategy to respond to the pages.

Our course added to the existing body of literature showing the benefits of simulation and experiential learning. Mock paging programs allow medical students the chance to practice one of the key roles of an intern in a low-risk environment and receive feedback on their management and communication skills. This activity can be a stand-alone program or can be incorporated into a larger course that helps prepare fourth-year medical students, and the variations allow for flexibility within a busy med school curriculum.

## Appendices

Cases with Checklists.docxPatient Sign-outs.docx
All appendices are peer reviewed as integral parts of the Original Publication.
