# Supplementary material for: A Paging Training Program for a Fourth-Year Internship Readiness Course
Source: MedEdPORTAL. 2020 Nov 13;16:11021. doi: 10.15766/mep_2374-8265.11021 (PMC7666833; doi:10.15766/mep_2374-8265.11021)
Supplement: Supplementary file 1 — Cases with Checklists.docxPatient Sign-outs.docx [file mep_2374-8265.11021-s001.zip › A. Cases with Checklists.docx]

**Case #1**

**CC:** SOB

**Additional communication challenge:** none

**PAGE:**  Urgent need action: 3134 w SOB, O2 sat 83%. NAME OF CALLER

**When student calls, evaluator should answer phone with:** “This is NAME.“

**If student does not exactly specify what they are calling about, evaluator should say:** “What are you calling in reference to?”

**If student does specify why they are calling, evaluator should state:**

“Mr. Wallace started to c/o SOB when I was helping him get to his chair a few moments ago.”

**If asked about specifically, the following should be provided. (If student asks for information you do not have, state “I do not have that information.”):**

| **Vitals:** | BP 138/86  HR 120  RR 40  T 37.2/99.0  O2 sats dropped to 83% on RA but came up to 96% with 3L of oxygen via nasal cannula. |
| --- | --- |
| **Is the patient having chest pain?** | No |
| **Assessment/How does he look?** | Alert and oriented, but appears uncomfortable  He is talking to me but having to stop to take breaths.  Lungs: CTAB. Breath sounds are present bilaterally.  CV: RRR, no MRG  Pulses normal |
| **Access** | He has 2 peripheral IVs |
| **Most recent CBC** | H/H 9.2/27 |
| **DVT prophylaxis** | He is not receiving any anticoagulation. He has not been wearing his SCDs. |

**Evaluation of phone call:**

| **Communication:** |  |
| --- | --- |
| Student identified themselves |  |
| Student identified reason for calling |  |
| Student asked specific, goal-directed questions |  |
| Student indicated they would call their senior resident |  |
| Student initiated read-back (closed-loop communication) |  |
| Student explained they were concerned about PE. |  |
|  |  |
| **Assessment and management:** |  |
| Student asked for vitals |  |
| Student asked about chest pain |  |
| Student asked for assessment of patient |  |
| Student asked about DVT prophylaxis |  |
| Student indicated they would come see the patient immediately |  |
| Student asked for the following: |  |
| Portable CXR |  |
| EKG |  |
| Cardiac enzymes |  |
| ABG |  |
| Student mentioned likely need for CTPA |  |
| Student did not send patient off the floor for chest x-ray |  |

**Comments:**

**Case #2**

**CC:** CP

**Additional communication challenge:** none

**PAGE:** Urgent need action: 4134 c/o CP. Please call back. NAME OF CALLER

**When student calls, evaluator should answer phone with:** “This is NAME.“

**If student does not exactly specify what they are calling about, evaluator should say:** “What are you calling in reference to?”

**If student does specify why they are calling, evaluator should state:**

“Mr. Brown’s wife called me in the room a few moments ago because he was having some chest pain. She is very upset.”

**If by the end of the call student does not specify that they will come talk to the wife as well, evaluator should ask:** “What should I do about his wife?”

**If asked about specifically, the following should be provided. (If student asks for information you do not have, state “I do not have that information.”):**

| **Vitals:** | BP 142/84  HR 98  RR 24  Temp 37.0/98.6  O2 sats 94% on RA |
| --- | --- |
| **Details about chest pain** | Pain is sharp and in his chest; radiates to jaw and left arm. Pain started 10 minutes ago. |
| **Did he receive nitro and did it help?** | He received 1 tab sublingually when the pain started with no relief of pain. |
| **Is he having any nausea/vomiting? Diaphoresis? Difficulty breathing?** | No nausea/vomiting  He is mildly sweating  He is having a slightly difficult time breathing. |
| **Access** | He has 2 peripheral IVs |
| **DVT prophylaxis** | Enoxaparin 80 mg twice daily |

**Evaluation of phone call:**

| **TASK** | **Student completed** |
| --- | --- |
|  |  |
| **Communication:** |  |
| Student identified themselves |  |
| Student identified reason for calling |  |
| Student asked specific, goal-directed questions |  |
| Student indicated they would call their senior resident |  |
| Student initiated read-back (closed-loop communication) |  |
| Student indicated to nurse they will talk to patient’s wife when they arrive (did *not* just tell nurse to deal with the wife) |  |
| **Assessment and management:** |  |
| Student asked for vitals |  |
| Student asked about chest pain (duration, radiation, etc.) |  |
| Student asked if the patient received nitro *and* if that relieved the pain (must ask about both) |  |
| Student asked about associated symptoms |  |
| Student indicated they would come see the patient immediately |  |
| Student mentioned the need for the following: |  |
| Portable CXR |  |
| EKG |  |
| Cardiac enzymes |  |
| Aspirin 325 |  |
|  |  |
| **TOTAL TASKS COMPLETED BY STUDENT** |  |

**Comments:**

**Case #3**

**CC:** fever

**Additional communication challenge:** none

**PAGE:** Urgent need action: 3134 febrile to 38.9. Please call back. NAME OF CALLER

**When student calls, evaluator should answer phone with:** “This is NAME.“

**If student does not exactly specify what they are calling about, evaluator should say:** “What are you calling in reference to?”

**If student does specify why they are calling, evaluator should state:**

“Mrs. Brown just spiked a fever to 38.9.”

**If asked about specifically, the following should be provided. (If student asks for information you do not have, state “I do not have that information.”):**

| **Vitals:** | BP 114/58  HR 100  RR 18  Temp 38.9C/102.0F  Sat 97% on room air |
| --- | --- |
| **Assessment** | Alert, but not oriented to person, place, or time (baseline – only specify if student asks if this is a change from baseline), mildly agitated  Lungs: Crackles bilaterally  Mildly tachycardic, strong pulses throughout  Abdomen: Soft, nontender, normal bowel sounds |
| **Access** | She has 2 peripheral IVs |

**Evaluation of phone call:**

| **TASK** | **Student completed** |
| --- | --- |
|  |  |
| **Communication:** |  |
| Student identified themselves |  |
| Student identified reason for calling |  |
| Student asked specific, goal-directed questions |  |
| Student indicated they would call their senior resident |  |
| Student initiated read-back (closed-loop communication) |  |
|  |  |
| **Assessment and management:** |  |
| Student asked for vitals |  |
| Student asked for assessment of patient |  |
| Student indicated they would come see the patient immediately |  |
| Student mentioned the need for the following: |  |
| Portable chest x-ray |  |
| Urine culture |  |
| Blood culture |  |
| CBC |  |
| Acetaminophen |  |
| Student did not order ibuprofen |  |
|  |  |
| **TOTAL TASKS COMPLETED BY STUDENT** |  |

**Comments:**

**Case #4**

**CC:** hypoxia

**Additional communication challenge:** none

**PAGE:** Urgent need action: 3134 w/ hypoxia. Please call back. NAME OF CALLER

**When student calls, evaluator should answer phone with:** “This is NAME.“

**If student does not exactly specify what they are calling about, evaluator should say:** “What are you calling in reference to?”

**If student does specify why they are calling, evaluator should state:**

“Ms. Brady’s O2 sats dropped to 87% on room air; she was placed on 4L of oxygen via nasal cannula, and her sats improved to 92%.”

**If asked about specifically, the following should be provided. (If student asks for information you do not have, state “I do not have that information.”):**

| **Vitals:** | BP 112/62  HR 96  RR 17  Temp 38 |
| --- | --- |
| **Is the patient having chest pain?** | No |
| **Assessment/How does she look?** | Drowsy appearing, having trouble talking to me  Rhonchorous breath sounds  Pulses normal |
| **Access** | She has 2 peripheral IVs |
| **DVT prophylaxis** | Lovenox |

**Evaluation of phone call:**

| **TASK** | **Student completed** |
| --- | --- |
|  |  |
| **Communication:** |  |
| Student identified themselves |  |
| Student identified reason for calling |  |
| Student asked specific, goal-directed questions |  |
| Student indicated they would call their senior resident |  |
| Student initiated read-back (closed-loop communication) |  |
| Student indicated possible transfer to ICU. |  |
|  |  |
| **Assessment and management:** |  |
| Student asked for vitals |  |
| Student asked about chest pain |  |
| Student asked for assessment of patient |  |
| Student asked about DVT prophylaxis |  |
| Student indicated they would come see the patient immediately |  |
| Student mentioned the need for the following: |  |
| Portable chest x-ray |  |
| EKG |  |
| Cardiac enzymes |  |
| ABG |  |
| Positive pressure, non-invasive ventilation |  |
| Student did not indicate that the patient should be immediately intubated. |  |
|  |  |
| **TOTAL TASKS COMPLETED BY STUDENT** |  |

**Comments:**

**Case #5**

**CC:** back pain

**Additional communication challenge:** The nurse is frustrated because they are very busy. Will answer all questions completely however.

**PAGE:** Urgent need action: 3134 c/o back pain. Please call back. NAME OF CALLER

**When student calls, evaluator should answer phone with:** “This is NAME.“

**If student does not exactly specify what they are calling about, evaluator should say:** “I have a lot going on right now; I’m not sure who you’re calling about.”

**If student does specify why they are calling, evaluator should state:**

“He is still complaining about his back pain, and he doesn’t have anything ordered for pain, and you know, I really don’t have time for this right now. I told the day team to order something, but I guess they forgot.”

**If asked about specifically, the following should be provided. (If student asks for information you do not have, state “I do not have that information.”):**

| **Vitals:** | BP 112/62  HR 96  RR 17  Temp 38 |
| --- | --- |
| **Tell me more about the back pain.** | It’s his chronic back pain, and he normally takes ibuprofen at home for it. |
| **Assessment/How does he look?** | Alert, oriented in no acute distress  Lungs: clear to auscultation bilaterally  CV: regular rate and rhythm, no murmurs  No neurologic deficits (*only provide if specifically asked for*) |
| **What did you ask the day team to order/what would you like?** | Tylenol |
| **Access** | He has 2 peripheral IVs |
| **DVT prophylaxis** | Heparin |

**Evaluation of phone call:**

| **TASK** | **Student completed** |
| --- | --- |
|  |  |
| **Communication:** |  |
| Student identified themselves |  |
| Student identified reason for calling |  |
| Student asked specific, goal-directed questions |  |
| Student initiated read-back (closed-loop communication) |  |
| Student did not argue with/interrupt nurse |  |
| Student empathized with nurse |  |
|  |  |
| **Assessment and management:** |  |
| Student asked for vitals |  |
| Student asked for more details about his back pain |  |
| Student asked for assessment of patient |  |
| Student indicated they would come see the patient if he continued to complain of back pain |  |
| Student ordered Tylenol |  |
| Student did not order back imaging |  |
|  |  |
| **TOTAL TASKS COMPLETED BY STUDENT** |  |

**Comments:**

**Case #6**

**CC:** Hypertension

**Additional communication challenge:** none

**PAGE:**  Urgent need action: 3134 BP 220/110. NAME OF CALLER

**When student calls, evaluator should answer phone with:** “This is NAME.“

**If student does not exactly specify what they are calling about, evaluator should say:** “What are you calling in reference to?”

**If student does specify why they are calling, evaluator should state:**

“Mr. Smith’s blood pressure was 200/110 on routine check.”

**If asked about specifically, the following should be provided. (If student asks for information you do not have, state “I do not have that information.”):**

| **Vitals:** | BP 200/110  HR 90  RR 16  T 37.0/98.6  O2 sats 97% on room air |
| --- | --- |
| **Was it a manual or machine reading?** | Machine |
| **What have their previous blood pressures been?** | 150s-160s/70s-80s |
| **Is the patient having chest pain?** | No |
| **Assessment/How does he look?** | Alert and oriented, appears comfortable  Breathing comfortably  Lungs: Clear to auscultation bilaterally.  CV: Regular rate and rhythm  Pulses normal |
| **Urine output?** | Normal (specify 70 cc/hr if specifically asked about) |
| **Has he been getting his home meds?** | Yes but is due for his next dose |
| **Access** | He has 2 peripheral IVs |

**Evaluation of phone call:**

| **TASK** | **Student completed** |
| --- | --- |
|  |  |
| **Communication:** |  |
| Student identified themselves |  |
| Student identified reason for calling |  |
| Student asked specific, goal-directed questions |  |
| Student initiated read-back (closed-loop communication) |  |
|  |  |
| **Assessment and management:** |  |
| Student asked for vitals |  |
| Student asked for previous blood pressure readings |  |
| Student asked about chest pain |  |
| Student asked for assessment of patient |  |
| Student asked about urine output |  |
| Student asked if it was a manual or machine reading |  |
| Student indicated to give patient his home meds and recheck blood pressure |  |
| Student did not immediately start a nitro drip, etc. |  |
|  |  |
| **TOTAL TASKS COMPLETED BY STUDENT** |  |

**Comments:**
