# Supplementary material for: A Paging Training Program for a Fourth-Year Internship Readiness Course
Source: MedEdPORTAL. 2020 Nov 13;16:11021. doi: 10.15766/mep_2374-8265.11021 (PMC7666833; doi:10.15766/mep_2374-8265.11021)
Supplement: Supplementary file 1 — Cases with Checklists.docxPatient Sign-outs.docx [file mep_2374-8265.11021-s001.zip › B. Patient Sign-Outs.docx]

Case #1:

Room #: 3134

Code status: FULL CODE

HD#5 for Mr. Wallace, a 68-year-old patient with PMH HTN and DM admitted to general medicine for anemia likely secondary to a GI bleed.

1. Anemia – Last H/H 9.5/28. Transfuse to goal Hgb 8
2. HTN – metop 25 mg BID, has been well controlled this admission
3. DM – holding home metformin. On SSI

To do:

[ ] F/u CBC 20:00

Case #2:

Room #: 4134

Code status: FULL CODE

HD#1 for Mr. Brown, a 67-year-old patient with hx of COPD, DM, CAD admitted to ACS for unstable angina.

1. UA/CAD –
   1. Supplemental O2 to maintain sats >90%
   2. Nitro sl prn for CP
   3. Metoprolol 25 mg BID
   4. Atorvastatin 80 mg daily
   5. ASA 81 mg daily, Plavix 75 mg daily
   6. Enoxaparin 80 mg BID
2. COPD – on home medications (Symbicort + Albuterol prn)
3. DM – holding home metformin, on SSI

To do:

[ ] Recheck Mg and K at midnight and replete as indicated

Case #3:

Room #: 3134

Code status: DNR/DNI

HD#3 for Mrs. Brown, a 79-year-old female w hx of dementia and CKD admitted to gen med from a local skilled nursing facility for encephalopathy of unclear etiology.

1. Encephalopathy – Minimize delirium precautions. Workup negative
2. CKD – Baseline creatinine 1.8. Avoid nephrotoxic agents.

Contingency:

-If she spikes a fever, obtain cultures

To do:

NTD

Case #4

Room #: 3134

Code status: FULL CODE

HD#3 for Ms. Brady, a 54-year-old female admitted to gen med for a COPD exacerbation.

1. COPD exacerbation – Azithromycin, Oxygen as needed to maintain O2 sats 92-95%, scheduled Duonebs

To do:

NTD

Case #5

Room #: 3134

Code status: FULL CODE

HD#2 for Mr. Jones, a 57-year-old male admitted to gen med for an AKI.

1. AKI – Cr 1.7 on admission, 1.5 this morning. Likely 2/2 NSAID use.

To do:

NTD

Case #6

Room #: 3134

Code status: FULL CODE

HD#1 for Mr. Smith, a 62-year-old male with hx of DM Type II, HTN, COPD admitted for a COPD exacerbation.

1. COPD exacerbation – Azithromycin, Oxygen as needed to maintain O2 sats 94-95%, scheduled Duonebs
2. HTN – on home Metoprolol 25 mg twice daily, Amlodipine 5 mg
3. DM – holding home Metformin, on SSI

To do:

NTD
